# N of 1 trials and the optimal individualisation of drug treatments: a systematic review protocol

**DOI:** 10.1186/s13643-017-0479-6

**Published:** 2017-04-24

**Authors:** Weyinmi A. Demeyin, Julia Frost, Obioha C. Ukoumunne, Simon Briscoe, Nicky Britten

**Affiliations:** 1 Institute of Health Research, University of Exeter Medical School, South Cloisters, St Luke’s Campus, Exeter, EX1 2LU UK; 2 NIHR CLAHRC South West Peninsula, University of Exeter Medical School, St Luke’s Campus, Exeter, EX1 2LU UK

**Keywords:** N of 1, ABAB, Multi-crossover, Individualised care, Within patient

## Abstract

**Background:**

Guidelines and evidence-based drug treatment recommendations are usually based on the results of clinical trials, which have limited generalisability in routine clinical settings due to their restrictive eligibility criteria. These trials are also conducted in ideal and rigorously controlled settings. N of 1 trials, which are single patient multiple crossover studies, offer a means of increasing the evidence base and individualising care for individuals in clinical practice. This systematic review of the N of 1 drug treatment trial aims to investigate its usefulness for achieving optimal individualised patient care.

**Methods:**

The following databases will be searched for relevant articles: MEDLINE, EMBASE, PsycINFO (all via Ovid), AMED, CINAHAL (via EBSCO), The Cochrane Library (including CENTRAL, NHS EED, and DARE), and Web of Science (Thomson Reuters). Supplementary searches will include ongoing trial databases and organisational websites. All N of 1 trials in which patients have been treated with a drug will be considered. Outcomes will include information on the clinical usefulness of N of 1 trials—i.e. achievement of optimal individualised care, health-care utilisation of patients, frequently used practices, experiences of clinical care or participation in N of 1 trials, adherence to treatment plan, and unwanted effects of the treatment. Screening of included papers will be undertaken independently by two reviewers, while data extraction and the quality of reporting will be conducted by one reviewer and checked by another. Both quantitative and qualitative summaries will be reported using appropriate methods.

**Discussion:**

This review will provide new insights into the clinical utility of N of 1 drug trials in helping participants find the most acceptable treatment as defined by patients and clinicians based on the selected outcome measures and the perspectives of participants involved in such trials. Findings from this review will inform the development of a stakeholder workshop and guidance to help physicians find the optimum therapy for their patients and will help guide future research on N of 1 trials.

**Systematic review registration:**

PROSPERO CRD42016032452

**Electronic supplementary material:**

The online version of this article (doi:10.1186/s13643-017-0479-6) contains supplementary material, which is available to authorized users.

## Background

Randomised controlled trials (RCTs) are considered to be the gold standard research method, but they have some limitations. One issue with RCTs relates to the applicability of the results to the individual patient. RCTs might exclude the elderly, children, ethnic groups, and participants with multiple conditions or rare diseases, thereby not only limiting the external validity of their results but also resulting in a paucity of evidence-based information for these groups. For example, in a review to assess the representativeness of RCT samples, it was concluded that they were not always seen as representative of the patients treated in routine clinical practice [1]. Furthermore, patients may react differently even when given the same drug for the same disease, a phenomenon termed heterogeneity of treatment effect (HTE). These differences between individuals may mean that the findings from RCTs are limited. RCTs only provide an estimate of the average treatment effect and are usually conducted in ideal settings. Therefore, they are not necessarily effective for all individual patients in real-world clinical practice.

These limitations could mean that clinicians are uncertain as to the effectiveness of a drug and might utilise a trial and error approach to guide treatment choice, sometimes called the *trial of therapy* [2]. A trial of therapy involves the provision of a single treatment to a patient, with the effectiveness of that treatment judged by the resultant clinical course. Apart from being unstandardised with no study protocols, trials of therapy are also susceptible to various problems, including the stability of the condition (current symptoms may subside with or without treatment), placebo effect, the desire of patients and doctors to please each other, their expectations about the treatment effect, regression to the mean since treatment effect is assessed once, and that only one treatment may be tested [3]. In the latter case, if the treatment is found effective the trial is ended with no further trials carried out on other treatments to determine if they are equally or even more effective than the first [2]. All of these factors could lead to false confidence in the primary therapeutic decision, as they all have an influence on the treatment outcome. Therefore, there is a need for a more methodologically rigorous approach to determining the treatment effect in the individual patient, for example, the N of trial.

In clinical medicine, the term N of 1 trial has been used to describe trials with multiple crossover and a repeat challenge-withdrawal design; for example, the ABAB design sequence in which a single patient receives an intervention during some periods (A) and then receives the control or alternate intervention or no intervention during the other periods (B) [4, 5]. In N of 1 trials in clinical medicine, individuals are studied during at least four periods—that is both the A and B phase are repeated at least once. Patients are switched sequentially from active drug to control (standard care and placebo) or between two or more active treatments, or between specific doses of a drug.

N of 1 trials could help to overcome some of the limitations of the other designs mentioned previously. The N of 1 trial is a study design that potentially lends itself more naturally than the RCT to individualising treatment [3], as individualised treatment effect estimates are generated rather than average treatment effect estimates. Furthermore, Willke and colleagues have suggested the use of N of 1 trials as a way of overcoming HTE, as the data obtained from these trials provide results that are applicable to the specific participating patients. In contrast to the unstandardised trials of therapy, N of 1 trials offer a more standardised approach for investigating the effectiveness of a drug. Furthermore, the use of multiple crossovers, blinding, and randomisation in this design ensures that results are less susceptible to regression to the mean, placebo effect, and other forms of bias that characterise trials of therapy. N of 1 trials could serve as a means of increasing the evidence base for people with similar characteristics to those who are typically excluded from clinical trials. A further advantage of the N of 1 design is that patients are usually involved in the selection of treatment for investigation, documentation of processes, and outcomes before the trial, as well as the selection of final treatment to be administered at the end of the trial. This promotes patient-centred care and patient engagement [4, 6]. Due to their higher applicability and validity for individual patients when compared to RCTs, N of 1 trials are judged to be of the highest strength in terms of clinical decision-making [7]. The Oxford Centre for Evidence-Based Medicine has also ranked N of 1 trials as “level 1” evidence for making treatment decisions in individual patients [8].

There are a number of situations where this design can be used effectively. Clinicians can use N of 1 trials for patients with chronic stable conditions who will be placed on long-term therapy and for whom the effectiveness of their current treatment is in doubt [2]. The trial design has been used widely in stable conditions affecting the nervous, respiratory, digestive or musculoskeletal systems, and in mental and behavioural disorders [9]. When applied to these conditions, it has been suggested that the drug under consideration should ideally have a rapid onset of action as shorter treatment periods make trial processes easier on the patient, although it may be possible to use drugs with a longer onset of action [2]. Guyatt and colleagues [2] also recommend that the drug should have a short duration of action to avoid unnecessarily long washout periods which could compromise the feasibility of the trial [2]. The duration of each period should take into account the time it takes for a drug to reach its full effect and the time required for this effect to subside completely. N of 1 trials are not for acute, or rapidly progressing diseases, but they have been used in conditions with episodic symptoms like seizures or migraines [2]. In these cases, the duration of each treatment period should be long enough for an attack or exacerbation to occur.

### Previous reviews of N of 1 trials

There have been two previous systematic reviews of N of 1 trials [10, 11], neither of which assessed the conditions for which N of 1 trials have been most useful or the different practices employed by investigators when conducting these trials. Punja et al. [9, 12] have published the results of the systematic review thesis by Punja [11]. Gabler and colleagues [10] provided an overview of N of 1 trials and aimed to determine the extent to which treatment decisions are influenced by N of 1 trial results and whether N of 1 trials provided sufficient data to conduct analyses that use a Bayesian framework for the purpose of estimating HTE or to obtain a more accurate estimate of treatment effect. They also explored whether treatment decisions are influenced by participation in an N of 1 trial, but did not investigate whether these varied with the condition of a patient. Punja [11] provided an overview of the methodology and reporting of N of 1 trials and sought to describe the statistical methods used to analyse data from N of 1 trials and how the results from several N of 1 trials could be combined. The author assessed how outcome measures from N of 1 trials conducted in participants with a similar condition assessing the same treatment can be aggregated to obtain average estimates of effect. They also assessed how these results can be combined across participants and studies, including RCTs, to obtain estimates of population-averaged treatment effect. Although studies comparing N of 1 trials to standard practice have been undertaken [13, 14], no review exploring the reported benefit of N of 1 trials in comparison to standard practice has been conducted. The previous reviews did not provide information on participants’ perspectives regarding their participation in N of 1 trials. The aim of this review is to determine the clinical conditions in which N of 1 trials have been shown to be effective in helping patients achieve an individualised treatment strategy. Furthermore, this review is looking to see if trials with patient-selected outcomes are more clinically useful than trials where patients do not select their outcomes and also, what influences patients’ choice of outcomes. Neither of the above reviews examined these aspects.

N of 1 trials often provide estimates of treatment effect for each drug treatment. This review is not concerned with the effectiveness of the therapies under investigation but rather it aims to ascertain whether the process of participation in an N of 1 trial is useful in helping the specific patient or clinician to identify an individualised drug treatment plan. This review will also examine typical practices in N of 1 trials and whether there is any change in a patient’s knowledge, attitude or treatment satisfaction after the trial. Findings from this systematic review will inform the development of a stakeholder workshop of clinicians and patients and a guidance which will assist clinicians to conduct N of 1 trials.

### Aims

The aims of the systematic review are to:

1. Provide a detailed typology of the N of 1 trial design.
2. Determine the particular challenges that N of 1 trials can address.
3. Identify the clinical conditions in which they are useful.
4. Investigate if the N of 1 trial is a clinically useful way of determining appropriate treatment for an individual patient.
5. Identify practices in N of 1 trials that could encourage patient participation.
6. Describe the experiences, views, and perceptions of participants in N of 1 trials and the factors guiding patients’ choice of outcomes.

Clinical usefulness will be determined by exploring the added benefit of N of 1 trials over standard practice and by analysing individual N of 1 trials. Measures of clinical utility will include, but not be limited to, the following: achievement of individualised care or trial objectives, identification of treatment responders and non-responders, and improvement in patients’ health care utilisation such as knowledge about their condition and self-management habits. As the review is also concerned with patient participation, the review will seek to identify practices that might encourage shared decision-making (e.g. patient selected outcomes, use of subjective outcome measures like patient diaries, and seeking the preferences of patients) or tailoring of drug treatment to a specific individual by using pre-trial dose finding. These examples were identified by reading through the key N of 1 papers [3, 15, 16].

### Research questions

1. What are the different design sequences of N of 1 trials?
2. What are the challenges that N of 1 trials can address?
3. In which clinical conditions are N of 1 trials more useful?
4. Are N of 1 trials more clinically useful compared to standard practice based on the above measures of clinical utility?
5. i. Are trials that encourage patient participation more clinically useful than trials that do not?
  ii. Do trials that encourage patient participation have a lower dropout rate?
6. What are the experiences, views, and perceptions of participants involved in an N of 1 trial and the factors guiding patients’ selection of outcomes?

## Methods

The Preferred Reporting Items for Systematic Reviews and Meta-Analysis Protocol (PRISMA-P) statement guides the reporting of this protocol and is included as Additional file 1 [17]. The PROSPERO International Prospective Register of Systematic Reviews, registration number is CRD42016032452.

### Eligibility criteria

#### Study design

Multiple crossover studies with a repeat challenge-withdrawal design (treatments are switched sequentially such as A-B-A-B or A-B-C-A-B-C) will be included. Trials with the sequence A-A-B-B or A-A-B-B-C-C will only be included if there is an assessed washout period in between periods that administer the same treatment. The order of allocation need not be randomised, but at least one of the treatments under investigation will be a drug, and studies should aim to determine the effect of a treatment for individual study participants. In the context of this review, a drug will be defined as any chemical substance or combination of this substance which may be used, for administration to a person, for the purpose of altering, correcting, or restoring physiological functions by exercising a metabolic, immunological, or pharmacological action [18].

#### Participants

All studies in human participants using the N of 1 trial design as defined above will be included in the review.

#### Comparator

Included trials should at least have one of the following comparators tested for within patients: placebo, or standard care, or alternative treatment or specific dose(s) of the same treatment. The washout period will be considered as a control period if an assessment is undertaken.

### Outcomes

The outcomes referred to below relate to the results of our study objectives, that is, the properties of the individual trials. The outcomes were chosen based on the Cochrane Consumers and Communication Review Group’s taxonomy [19], outcomes of relevance to end-users with regards to effective medicine use [20], reviews on enhancement of medication adherence [21], and individualised care planning [22].

Outcomes will include:

1. In terms of aim 1, the identification of different study types and their categorisation.
2. For aim 2, the identification of the clinical challenges addressed and their categorisation.
3. The identification of the clinical conditions for which N of 1 trials have been conducted and their categorisation.
4. Measures of clinical usefulness for aim (4) will include the following: trial enabled participants to find the most acceptable treatment or allowed trial objectives to be met based on the patient, clinician or researcher’s judgement. Other measures of usefulness will include identification of treatment responders and non-responders, improvement in patients’ health care utilisation, knowledge and understanding about their condition, treatment satisfaction, post-trial preference, self-management practices, self-efficacy, and ability to access support and/or information, confidence, and competence.
5. For aim (5), typical practices in N of 1 trials that could allow patients to participate actively in the research process and decision-making—that is elements of shared decision-making or pre-trial dose finding. These may include patients selecting their outcomes, use of subjective health and wellbeing measures, and use of objective health and wellbeing measures. Data on dropout rates will also be included.
6. For aim (6), outcomes will include participants’ experiences, views, and perceptions about N of 1 trials.

### Exclusion criteria

We will exclude studies without a multiple crossover design or without a repeat challenge-withdrawal design (such as A-B, or A-B-C or A-B-A, or A-B-A-C-A-D) and trials with a crossover design involving simultaneous treatment on both sides of the body. Molecular/genetic studies, study protocols, methodological papers, reviews, and studies which aim to prevent or determine causation of a disease or in which a drug was not among the treatments will also be excluded.

### Search strategy

The following biomedical electronic databases will be searched for relevant articles: MEDLINE, EMBASE, PsycINFO (all via Ovid), AMED, CINAHAL (via EBSCO), The Cochrane Library (including CENTRAL, NHS EED, and DARE), and Web of Science (Thomson Reuters). A draft search strategy developed in MEDLINE has identified approximately 2000 abstracts (see Additional file 2). This will be adapted for use in the other databases and grey literature resources. The search strategy, developed with the help of an information specialist (SB) includes terms for N of 1 combined using AND with terms for randomised controlled trial and individualised care, with no limitation in dates.

The authors are aware that some N of 1 studies are present in the grey literature; therefore, the following sources will be searched: Controlled Clinical Trials, ClinicalTrials.gov, International Clinical Trials Registry Platform (ICTRP), and Epitemonikos (a systematic review database). Organisational websites will also be searched: World Health Organization, UK Department of Health, U.S. Department of Health and Human Services, American College of Clinical Pharmacology, American Society for Clinical Pharmacology and Therapeutics, British Pharmacological Society, Australian Department of Health and Ageing and Health Canada. Additional papers will be identified by citation tracking, reference list checking and contacting authors of relevant articles. All searches will be conducted by one reviewer (WD).

### Study selection process

Results of all searches will be exported into Endnote X7 where duplicates identified automatically will be moved to a separate folder and reported in the final flow diagram. WD will also hand search for additional duplicates not previously identified by the software. Inter-rater agreement will be tested prior to the commencement of the screening process. All titles and abstracts identified from the search will be screened independently by two reviewers based on the eligibility criteria. All full text articles of potentially relevant papers will be retrieved and screened independently by two reviewers based on the same criteria. Discrepancies during the screening process will be resolved through discussion and where consensus cannot be achieved, a third reviewer will be consulted.

### Quality assessment

Assessment of study quality of all included papers will be undertaken by WD and checked for accuracy by another reviewer. Currently, there are no quality assessment tools for the appraisal of N of 1 trials in clinical medicine. A modified version of the CONSORT extension for reporting N of 1 Trials (CENT) [5] will be used to appraise the quantitative papers. This will be in a yes/no format to determine if the studies reported items in the checklist. Consolidated criteria for reporting qualitative research (COREQ) [23] and CENT will be modified and incorporated for the assessment of qualitative papers. Any discrepancies will be discussed and a third reviewer involved when necessary.

### Data extraction

A data extraction form will be developed using a modified version of the Consumers and Communication Review Group’s Data Extraction Template [19]. Data will be extracted independently by WD and checked for accuracy by another reviewer. The completed forms will be compared and disagreements discussed with reference to the original paper. A third reviewer will be consulted if a consensus cannot be reached. Authors of included N of 1 studies will be contacted and asked to provide missing data or clarify any ambiguities. If authors are unable to provide relevant information on any areas of uncertainty (due to missing data or missing details) in the trial reports, this will be noted.

Data will be extracted on the following: *Study methods*- aims and purpose of the study, N of 1 study design subtype, funding source, competing interests, and trial coordinator; *participant information*- country, setting, number of participants, characteristics of participants, and nature of the condition; *intervention details*- type of intervention, selection of intervention, procedure, intervention provider, frequency of intervention, comparator, presence or absence of washout, and fidelity; *outcomes*- all outcomes regarding clinical usefulness and typical practices as stated above, selection of outcome (by whom), method of assessment, timing of measurement, and dropout rates; *themes-* relating to participant views, satisfaction, perception, and attitude towards N of 1 trials will be extracted; patients’ preferences, experiences of being in an N of 1 trial, professional opinion about its relevance, factors guiding patient’s choice of an outcome, other qualitative data identified in the studies and relevant to the review question will also be extracted.

### Data analysis

Depending on the nature of the data extracted, quantitative data will be analysed descriptively. The first, second, and third aims of the review, i.e. typology of N of 1 trials based on the design sequences, particular challenges that they address and conditions in which they have been useful will be identified from the studies. They will be grouped and presented descriptively using numbers and percentages for categorical variables and means and standard deviations or medians and interquartile ranges for continuous data. It might further be presented in a graphical form to enable mapping of the typology to conditions in which they have been useful. The fourth aim, which is the clinical usefulness of N of 1 trials as stated above, will also be identified and reported. The fifth aim, typical practices in N of 1 trials as judged by the inclusion of elements of shared decision-making or pre-trial dose finding will be summarised descriptively. Trials with the stated practices will be compared to trials without them to ascertain if there are any differences in the clinical usefulness and results will be presented using percentages.

For the qualitative data, about participants’ experiences of N of 1 trials, the primary method of synthesis might be meta-ethnography if appropriate [24]. Meta-ethnography will be judged as appropriate if there are sufficient papers containing conceptually rich and detailed qualitative data to enable translation of studies into one another [25]. A summary of each paper will be constructed and diagramming or mapping may also help to organise and interpret data in the initial phases. This involves presenting information in a graphical form in order to show the relationship between the data [26]. The key themes and concepts identified in these studies will form the basis for the synthesis. Variability in accounts due to any of the following elements will be investigated: study design, participant’s condition, and methodology. Our analysis will be led by the study findings themselves, but we also anticipate that analysis will be informed by the findings in the quantitative synthesis to assess similarities, links, and differences between the two bodies of literature.

If it is not possible to summarise the data in the manner stated above, a narrative approach will potentially be used to summarise the quantitative and qualitative data into a coherent whole [27].

## Discussion

The N of 1 trial design offers a means of accurately determining the right medications for end users by tailoring treatment to individual needs, thus ensuring judicious use of scarce resources. This systematic review will examine the usefulness of the N of 1 trial as an intervention for individualising drug treatments in patients. We are interested in exploring how drugs can be adapted to suit the individual patient, both socially and medically. Although there have been two previous reviews about other aspects of N of 1 trials, to our knowledge, no other review has addressed this objective.

Poor quality of some of the studies may bias our results, and it is also possible that some N of 1 studies will be missed due to poor reporting and lack of uniformity in the terms used to describe such trials. We hope to identify as many relevant papers as possible by contacting authors for literature on N of 1 trials. We have already developed a comprehensive search strategy and tested it against the studies included in the Gabler review. Multiple reviewers will be involved at every stage of the review, which will help to strengthen the quality of the review. The findings from this review will inform the development of a stakeholder workshop and guidance to assist clinicians in conducting N of 1 trials.

## Additional files

Additional file 1: Preferred Reporting Items for Systematic Reviews and Meta-analysis Protocols (PRISMA-P) 2015 checklist: recommended items to address in a systematic review protocol. (DOCX 36 kb)

Additional file 2: Search strategy. Ovid SP MEDLINE search strategy. (DOCX 13 kb)

## Abbreviations

CENT: CONSORT extension for reporting N of 1 trials
HTE: Heterogeneity of treatment effect
RCT: Randomised controlled trial
