# Supplementary material for: N of 1 trials and the optimal individualisation of drug treatments: a systematic review protocol
Source: Syst Rev. 2017 Apr 24;6:90. doi: 10.1186/s13643-017-0479-6 (PMC5402671; doi:10.1186/s13643-017-0479-6)
Supplement: Supplementary file 2 — Search strategy. Ovid SP MEDLINE search strategy. (DOCX 13 kb) [file 13643_2017_479_MOESM2_ESM.docx]

**Additional file 2**

Database: MEDLINE

Host: OVID

Data Parameters: 1946 to September Week 2 2015

Date Searched: 18/09/2015

Searcher: WD

Hits: 2307

Strategy:

1. ('n-of-1' adj2 (analys?s or characteristic* or design or double-blind* or method* or randomi?ed or RCT* or stud* or treatment* or trial*)).tw.

2. (("single case*" or "single participant*" or "single patient*" or "single subject*") adj2 (design* or randomi?ed or trial* or stud*)).tw.

3. (("individual case*" or "individual patient*" or "individual subject*") adj2 (trial* or stud*)).tw.

4. (individual adj2 "double blind*").tw.

5. ('within-individual' adj2 (analys?s or trial* or stud*)).tw.

6. ('within-patient' adj2 (analys?s or trial* or stud*)).tw.

7. or/1-6

8. ((single or individual) adj (patient* or subject* or participant*)).tw.

9. Individualized Medicine/

10. ((individuali?ed or personali?ed) adj (treatment* or medicine*)).tw.

11. Patient-Centered Care/

12. ('patient cent*' adj (approach* or intervention*)).tw.

13. (individual* adj (approach* or need* or preference*)).tw.

14. or/8-13

15. Controlled Clinical Trial/

16. Clinical Trial/

17. Clinical Trials as Topic/

18. clinical trial*.tw.

19. Cross-Over Studies/

20. multiple cross over stud*.tw.

21. Randomized Controlled Trial/

22. Randomized Controlled Trials as Topic/

23. ("randomi?ed controlled" adj (stud* or trial*)).tw.

24. randomi?ed controlled trial.pt.

25. RCT*.tw.

26. Double-Blind Method/

27. or/15-26

28. 14 or 27

29. 7 and 28
